# Thermally Initiated Structural Transformations in the Temperature Range (624–643) ± 1 K for Amorphous Metal Alloy Al_87_Y_4_Gd_1_Ni_8_ and Influence on Mechanical Properties

**DOI:** 10.3390/ma19153194

**Published:** 2026-07-27

**Authors:** Khrystyna Khrushchyk, Paweł Świec, Yurii Kulyk, Krzysztof Aniołek, Vasyl Kordan, Małgorzata Karolus, Lidiya Boichyshyn

**Affiliations:** 1 Department of Physical and Colloidal Chemistry, Faculty of Chemistry, Ivan Franko National University of Lviv, 6 Kyryla and Mefodiia Str., 79005 Lviv, Ukraine; khrystyna.khrushchyk@us.edu.pl (K.K.); lidiya.boichyshyn@lnu.edu.ua (L.B.); 2 Faculty of Science and Technology, Institute of Materials Engineering, University of Silesia in Katowice, 1A Pułku Piechoty Str., 41500 Chorzów, Poland; pawel.swiec@us.edu.pl (P.Ś.); krzysztof.aniolek@us.edu.pl (K.A.); 3 Department of Inorganic Chemistry, Faculty of Chemistry, Ivan Franko National University of Lviv, 6 Kyryla and Mefodiia Str., 79005 Lviv, Ukraine; vasyl.kordan@lnu.edu.ua

**Keywords:** amorphous metal alloy (AMA) based on aluminum, crystallization, energy of activation, microhardness

## Abstract

**Highlights:**

- The activation energy of the nucleation, growth and stable crystallization processes calculated for AMA Al_87_Y_4_Gd_1_Ni_8_ equals to 193 ± 12, 199 ± 30, and 188 ± 18 kJ/mol and the frequency factor (k_0_) equals to 2.0 × 10^12^, 3.53 × 10^13^, and 4.41 × 10^11^ s^ −1^, respectively.
- It has been established that during isothermal annealing in the temperature range of 624–643 K, a thermally stable compound Al_19_Ni_5_(Y,Gd)_3_ is formed.
- Mechanical properties are improved by five and nine times after annealing at the temperatures of nucleation (T_1_ = 624 ± 1 K) and crystal growth (T_2_ = 633 ± 1 K) compared to the initial state of the amorphous metal sample.

**Abstract:**

Amorphous metal alloys (AMAs) are metastable materials that are characterized by good mechanical properties and corrosion properties. It is known that with certain thermal modifications, these properties improve or lose their value. The purpose of this research work is to investigate the optimal conditions of thermal modification that improve the mechanical properties of this alloy. The DSC method established the temperatures of phase transitions in the temperature range of 624–643 K, which correspond to the following processes: crystal nucleation (T_1_ = 624 ± 1 K), growth (T_2_ = 633 ± 1 K), and stable crystallization (T_3_ = 643 ± 1 K). The XRD and TEM/HREM methods revealed structural changes in the amorphous matrix as a result of thermal modification. As a result of isothermal annealing for 2 min. at temperatures T_1_, T_2_, T_3_, a solid solution based on aluminum and a thermally stable compound Al_19_Ni_5_(Y,Gd)_3_ were formed. The equation for the transformation of an amorphous matrix AMA Al_87_Y_4_Gd_1_Ni_8_ in the temperature range (624–643) ± 1 K during isothermal 2 min annealing is given by: Am → Am′_resid_+ solid solution Al(X) → Am′_(enriched REE)_ + solid solution Al(X) + nano-Al_19_Ni_5_(Y,Gd)_3_ → Am′_(enriched REE)_ + solid solution Al(X) + nano-Al_19_Ni_5_(Y,Gd)_3_. The Oliver–Pharr method established that AMAs annealed at temperatures T_1_ and T_2_ have microhardness indicators 5–9 times higher than amorphous samples; however, the material loses its elasticity under such thermal deformation conditions.

## 1. Introduction

### 1.1. Types of Crystallization

Amorphous metal alloys (AMAs) constitute a special class of metastable materials [1]. Unlike crystalline alloys, where atoms are arranged in a periodic lattice, only short-range order is preserved in the amorphous state [1,2,3,4]. Their free energy is higher than that of the corresponding crystalline phases, which causes a tendency to crystallization during heat treatment [4,5]. This property allows changing the physicochemical characteristics of the material in a controlled manner, forming a partially or completely nanocrystalline structure, for example α-Al [5,6,7]. Such structural transformations affect the electrochemical activity and corrosion behavior of the alloy, in particular, they lead to changes in the corrosion potential and mechanical properties [8,9]. This feature selects unique physical and chemical and mechanical properties of AMAs, which are significantly different from crystalline analogs [1,2,5,6].

Analysis of the scientific literature [9,10,11,12,13,14] indicates that AMAs of the Al–(Y,Gd)–Ni and Al–(Y,Gd)–Ni–Fe systems are of significant scientific interest due to the combination of high mechanical, thermal and corrosion properties. AMAs’ structure is characterized by the absence of long-range order, which ensures thermodynamic metastability and causes complex crystallization kinetics. Crystallization of AMAs can occur [15,16,17,18,19,20,21] under the influence of (i) temperature [15,16,17,18,19,20,21], (ii) mechanical load [15,17,20] or (iii) pressure [16,18].

(i) The temperature limits of crystallization depend on the composition of the AMA, the heating rate, and the energy barrier of the transition [7,8,9,10,11,12,13,14,15]. Thermal crystallization is the most common method for analyzing the stability of the amorphous state [1,6,8,10]. This type of crystallization occurs when heated above the glass transition temperature (Tg), at which atomic mobility increases and the nucleation of a crystalline phase begins. Thermal crystallization usually occurs in two stages:
  - Primary crystallization, accompanied by the formation of nanocrystalline nuclei (phases with grain sizes of ~5–20 nm);
  - Secondary crystallization, during which grain growth and coalescence occur, forming stable/metastable phases.
(ii) This type of crystallization is local in nature: crystal nuclei may appear in the deformation zone and grow due to mechanical energy. Depending on the intensity of the load and the temperature of the environment, this process may be reversible or lead to a complete transformation of the structure.
(iii) The main mechanism is the lowering of the energy barrier for the nucleation of a new phase under the influence of pressure, which enhances diffusion processes even at relatively low temperatures.

### 1.2. Influence of Alloying Elements in Al–TM–REM Systems on Crystallization Processes

Alloying is a key factor for the formation of the amorphous state of aluminum alloys, since pure aluminum is characterized by low glass-forming ability and a high tendency to crystallization even during ultrafast cooling [5,21].

The alloying of transition (TM) and rare earth (REEs) elements significantly changes both the thermodynamic and kinetic conditions of crystallization, ensuring the stabilization of the amorphous structure [5,9,22,23]. Such alloying allows obtaining materials with unique physicochemical properties that cannot be achieved in pure aluminum or in crystalline alloys [2,6,9,22]. Transition metals (nickel and iron) have a key role in Al–TM–REM systems [6,9]. Doping leads to increased chemical heterogeneity of the melt, increased viscosity, and reduced diffusion mobility of atoms [5,6]. These effects significantly complicate the processes of nucleation and growth of crystalline phases, which is critically important for preserving the amorphous state during rapid cooling [6,23]. In addition, partially filled d-orbitals of Ni and Fe affect the electronic structure of the alloy, increasing its surface activity and promoting the formation of active centers for electrochemical reactions [5,23]. Nickel not only promotes amorphization by increasing the glass formation ability (GFA) [8,11], but also increases the electrochemical activity of the alloy, in particular in cathodic hydrogen evolution reactions [24,25,26]. Iron, in addition, enhances this effect by creating catalytically active sites and influencing the kinetics of electrode processes, which ensures the overall efficiency of reactions on the AMA surface due to the synergistic interaction of components in the amorphous matrix [2,26].

The combination of Ni and Fe in the alloy composition achieves a synergistic effect, which simultaneously increases the stability of the amorphous state and surface activity [9,26]. In particular, partial replacement of nickel with iron slows down the diffusion of aluminum atoms, which shifts the nanocrystallization temperatures to higher values, increasing the thermal stability of the material [6]. Such alloys are convenient model objects for studying the dependence of electrochemical activity on composition and heat treatment, since the amorphous structure is devoid of traditional defects (grain boundaries, dislocations), which allows us to clearly trace the effect of alloying [2,5,9]. Transition metals simultaneously affect the structural characteristics (ability to form glass) and the electronic structure of the alloy, changing the density of states in d-orbitals and lowering the energy barrier of the hydrogen evolution reaction [5]. Rare earth elements (REEs), particularly yttrium and gadolinium, play a predominantly structure-forming role in amorphous aluminum alloys [5,6,9]. Their large atomic radii and significant electronegativities relative to aluminum lead to local distortion of the atomic environment, which effectively suppresses crystallization [5,6,7]. This effect allows the expansion of the temperature range of the existence of the amorphous state and increases in the thermal stability of alloys [5,7]. These properties are particularly important for further electrochemical and thermal studies, since a stable amorphous state provides a more predictable behavior of the material by promoting a uniform distribution of active centers [8,9]. In addition to their structural role, REEs can significantly modify the physicochemical properties of the surface of alloys [9]. Rare earth elements perform a dual task: they contribute to the structural stability of the amorphous state (increasing the ability to form glass) and regulate the electrochemical properties of the surface [5,6,9].

The combination/replacement of transition and rare earth elements in Al–TM–REM systems creates a synergistic effect, achieving a balance between high glass-forming ability, thermal stability, and electrochemical activity [5,8,9]. This allows the formation of alloys that combine a homogeneous disordered structure with the presence of numerous active centers on the surface, which ensures the rapid formation of protective films and high reactivity in alkaline environments [3,8,18]. The absence of traditional crystalline defects makes such materials ideal model objects for studying the relationship between composition, structure, and electrochemical behavior [3,8].

Of particular interest in this context are alloys doped simultaneously with Ni, Fe, Y, and Gd [6]. Such a combination of elements allows us to expect a simultaneous increase in the stability of the amorphous state (in particular, due to the slowing down of the diffusion of atoms by iron) [6] and an intensification of electrochemical processes in alkaline electrolytes [27].

Alloying with iron atoms performs a dual role: they increase the thermal stability of the amorphous phase, but can also activate the crystallization process, which is important for controlling the structure of the material after annealing. As a result of doping, not only the crystallization temperature changes, but also the entire kinetic process, which must be taken into account when designing materials with controlled properties. Table 1 compares the influence of iron atoms on phase transition temperatures, activation energies, and identifiable phases [27,28,29,30,31,32,33,34,35] formed in the amorphous matrix after crystallization processes.

Amorphous metal alloys based on aluminium solid solution can be precipitated in the processes of hydrogen evolution process [5] and wastewater treatment [36].

These articles [1,6,8,10] investigate the effect of one-hour annealing on the physical and chemical properties of aluminum-based AMA.

The scientific novelty of the work lies in the systematic study of short-term isothermal annealing (2 min) of the amorphous alloy Al_87_Y_4_Gd_1_Ni_8_ in the temperature range of (624–643) ± 1 K, which corresponds to three separate stages of crystallization. For the first time, the relationship between the activation energies of phase transformations, the formation of the phase composition (Al + Al_19_Ni_5_(Y,Gd)_3_) and the mechanical characteristics of the material has been established for this system. It is shown that selective activation of the early stages of crystallization provides significant strengthening of the material, while the transition to the stage of stable crystallization is accompanied by the degradation of properties.

The purpose of this work is to establish the regularities of structural transformations in the amorphous alloy Al_87_Y_4_Gd_1_Ni_8_ during short-term isothermal annealing at temperatures T_1_ = 624 ± 1 K, T_2_ = 633 ± 1 K, and T_3_ = 643 ± 1 K, as well as to determine their influence on the mechanical properties of the material with the determination of the optimal structural state.

## 2. Materials

The objects of the investigations were AMAs based on aluminum with the following composition: Al_87_Y_4_Gd_1_Ni_8_ in ribbon form. The thickness and width of the AMAs were 20–25 μm and 3 mm, respectively. The AMAs were obtained at the G. V. Kurdyumov Institute for Metal Physics of the Ukrainian Academy of Sciences (Kyiv) by the melt spinning method in a helium atmosphere on a copper drum rotating at a speed of ~30 m/s. The melt was prepared from pure metals and binary compounds REAl_3_ (RE = Y, Gd). The purity of the metals was as follows: Al (99.999 wt.%), Ni (99.99 wt.%), Y (99.96 wt.%), Gd (99.96 wt.%) and Fe (99.99 wt.%).

## 3. Methods of Investigation

### 3.1. Differential Scanning Calorimetry Analysis

The thermal properties of the samples were analyzed using a PerkinElmer Pyris 1 (710 Bridgeport Ave, Shelton, CT, USA). Using a DSC instrument under an argon atmosphere, heating was performed from room temperature to 900 K at rates of 10, 15, 20 K/min. The weight of the amorphous alloy was 1 g. Crystallization temperatures were determined from the positions of the exothermic peaks. The activation energy (E_a_) of the crystallization processes were estimated using the Augis–Bennett method.

The equation for calculating the activation energy using the Augis–Bennett method is as follows: $\;ln\frac{\beta }{T_{p}}=\;-\frac{E_{a}}{{RT}_{p}}+const$, where β—heating rate (K/min); E_a_—activation energy (J/mol); R—universal gas constant (8.314 J/mol·K); T_p_—peak temperature (K) [36,37].

### 3.2. X-Ray Diffraction Analysis

Phase analysis was performed by X-ray diffraction (XRD) (PANalytical, Almelo, The Netherlands) using a PANalytical Empyrean Diffractometer with Cu-Kα radiation (λ Kα1 = 1.5418 Å) and a PIXcelldetector. The XRD parameters were as follows: 2θ range from 20 to 120/130°, step size 0.03°, scan speed 1° per minute. Phase analysis was done based on the HighScore Plus PANalytical software (version 3.0) integrated with the ICDD PDF4 + 2025 crystallographic database. The Ehrenfest formula [3,38] was used to determine the average interatomic distance by the angular position of the diffraction maxima of the amorphous alloy. The Lorentz function was used to describe the diffraction line profile of crystalline phases. The angular dependence of the half-width of the maxima was approximated by the formula [3,38]: FWHM = $\frac{LX}{cos(\theta )}$, where LX is the dimensional parameter.

To determine the average sizes of the nanograins of the phase components, the following ratio was used [38,39]: L = $\frac{18}{3.14}\frac{\lambda }{\left(LX-LX_{e}\right)}$, where λ = 0.15148 nm—the wavelength of X-rays; LX, LX_e_—the size parameters of the studied and reference samples. The Al_87_Ni_8_Gd_5_ alloy, annealed at T = 415 °C for 2 h, was used as the reference sample.

Diamond Version 2.1d was used to visualize crystal lattices.

### 3.3. Transmission Electron Microscopy/High-Resolution Electron Microscopy Analysis

Microstructural investigations were performed using a JEOL JEM-3010 (Tokyo, Japan) high-resolution transmission electron microscope (HRTEM) operated at 300 kV. TEM specimens were prepared by cutting the metal strip to the appropriate size with a precision knife, followed by ion polishing using a GATAN 691 Precision Ion Polishing System at an ion acceleration voltage of 4 keV.

### 3.4. Scanning Electron Microscopy/Energy-Dispersive X-Ray Spectroscopy Analyses

The AMA surface was studied using a Tescan Vega 3 LMU scanning electron microscope (TESCAN GROUP, Brno, Czech Republic) and by energy-dispersive X-ray spectroscopy using an Aztec ONE microanalyzer with Si-shifted X-MaxN20 detector (20–25 kV voltage and vacuum regime; Oxford Instruments NanoAnalysis, High Wycombe, UK).

### 3.5. Mechanical Analysis

The mechanical properties of the AMAs were investigated using a Micro Combi Tester (Micro Scratch + Microindentation) MCT3 (Oliver–Pharr method [39]; type of indentor: Berkovich; material of indentor: diamond; indentation parameters: max load: 100.00 mN; loading/unloading rate: 200.00 mN/min; pause: 10.0 s) and ISO 14577 [40] for metallic materials—an instrumented indentation test for hardness and materials parameters (Anton Paar, Corcelles-Cormondrèche, Switzerland). The Young’s modulus (E_it_) calculated from E* using an estimated sample Poisson’s ratio (ν_s_) for metal was from 0.2 to 0.4. E_it_ = E* · (1 $-\;\nu_{s}^{2}$), where E* is calculated from following equation: E* = $1/\frac{1}{E_{r}}\;-\;\frac{1-\nu_{i}^{2}}{E_{i}}$, with E_i_ -Elastic modulus of the indenter (diamond 1141 GPa, ν_i_—Poisson’s ratio of the indenter (diamond 0.07)). The microhardness was calculated from the equation (modified Berkovich indenter): HM = $\frac{F}{A_{s}(h)}\;$≈$\;\frac{F}{26.97\cdot h^{2}}$, F in N, the time for the application of F in sec, the time during F_max_ is kept constant in sec. The elastic part of indentation work was calculated from the equation: η_IT_ = $\frac{W_{elast}}{W_{total}}$ · 100%, with W_total_ = W_elast_ + W_plast_. Plastic part W_plast_/W_total_ follows as 100%-η_IT_ (ISO/DIS Standart 14577-1:2015 [40], DIN Standart 50359-1 [41]). Five indentations were made on each sample. Static tensile testing was carried out on an INSTRON 5982 testing machine (Norwood, MA, USA). The length of the sample was 5 cm. The traverse speed of the machine during testing was 1 mm/min. Tensile curves were recorded in the coordinate system: stress σ—relative strain ε.

## 4. Results and Discussion

### 4.1. Kinetics of the Crystallization Process and X-Ray Analysis of Phases Transmition Within the Temperature Range 624–643 ± 2 K

**Figure 1:**
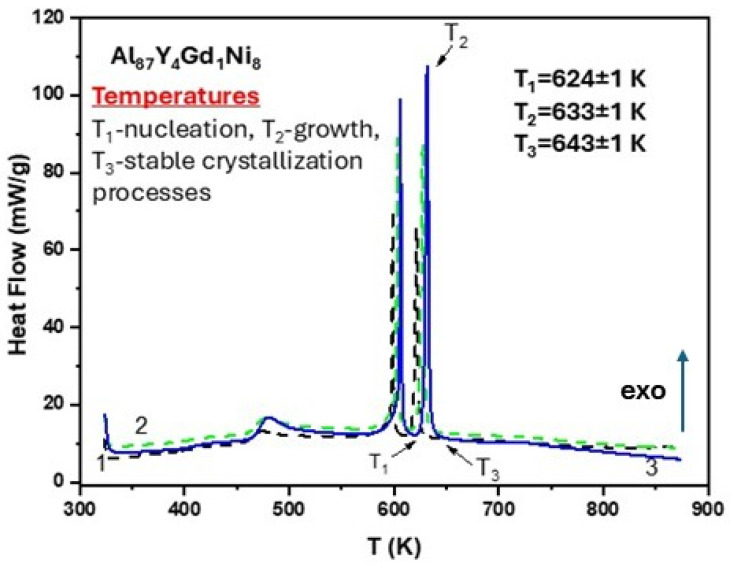
DSC curves with different heating rates (β): 1: 10 K/min—black line; 2: 15 K/min—green line; 3: 20 K/min—blue line for AMA Al_87_Y_4_Gd_1_Ni_8_.

Three phase transitions can be observed on the DSC curve (Figure 1): the first in the temperature range (448–495) ±1 K, the second in the temperature range (59–611) ± 1 K and the third in the temperature range (624–643) ±1 K at a heating rate of 20 K/min.

From the DSC curves (Figure 1), the phase transition temperatures were established within the third maximum at a heating rate of 20 K/min, which are as follows for AMA Al_87_Y_4_Gd_1_Ni_8_ temperatures T_1_ = 624 ± 1 K, T_2_ = 633 ± 1 K, and T_3_ = 643 ± 1 K.

Based on the DSC results at different heating rates, the kinetic parameters of the three stages of the structuring process were calculated: T_1_—nucleation of the nanocrystalline (crystalline) phase; T_2_—the stage at which, alongside the nucleation of nanocrystals, the growth of existing ones takes place; and T_3_—the stage at which the formation of new nuclei is practically absent and only the growth of existing nuclei at a constant rate occurs.

Using the Augis–Bennett kinetic models, the activation energy of the nucleation, growth and stable crystallization processes and the frequency factor (k_0_) were calculated. For AMA Al_87_Y_4_Gd_1_Ni_8_ the activation energy of the nucleation, growth and stable crystallization processes equals to 193 ± 12, 199 ± 30, and 188 ± 18 kJ/mol and the frequency factor equals to 2.0 × 10^12^, 3.53 × 10^13^, and 4.41 × 10^11^ s^−1^, respectively.

**Figure 2:**
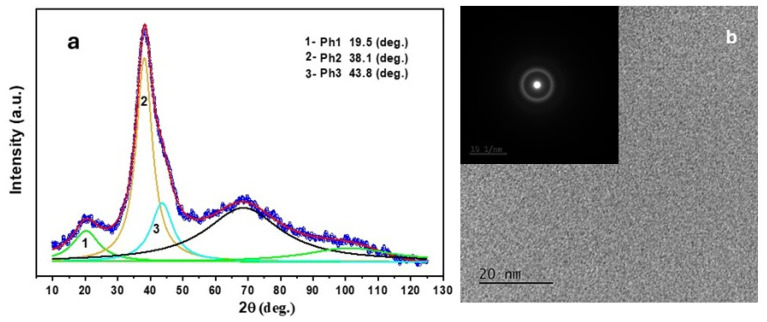
(**a**) The scattering intensity curve and (**b**) HREM images for the initial amorphous alloy Al_87_Y_4_Gd_1_Ni_8_.

In our previous work [7] we determined the activation energies for the crystallization process of the second phase transformation, which showed that for the Al_87_Gd_5_Ni_4_Fe_4_ alloy, the activation energy of the nucleation process is 357 ± 57 kJ/mol and for stable crystallization is 339 ± 5 kJ/mol; there are significant changes in the energy barriers required for phase transformations in the amorphous matrix. For the Al_87_Y_4_Gd_1_Ni_4_Fe_4_ alloy, the activation energy for the nucleation, growth and stable crystallization process practically does not change; the difference is 18 ± 6 kJ/mol. For the phase transition in the temperature range (624–643) ± 1 K, the activation energy for the processes of nucleation, growth and stable crystallization practically does not change and is ±5 kJ/mol, which indicates that the stability of the phases formed during the crystallization process frequency factor (k_0_), which indicates the maximum possibility of crystallite formation in an amorphous matrix, does not change significantly. The results of the crystallization kinetics correlate with the results of the X-ray structural analysis (Figure 2 and Table 2). The results of the XRD and HREM images in Figure 2 confirms the amorphous state of the alloy.

In the region of scattering angles 2θ ≈ 20° an additional maximum is observed, which indicates the presence of ordering of atoms of medium radius of action in the amorphous alloy along with the ordering of the nearest neighbors. In our previous publication [42] the same tendency is shown for the AMA Al_87_Y_4_Gd_1_Ni_4_Fe_4_, but with a more intensely pronounced peak in the region of small scattering angles (2θ ≈ 20°). It can be assumed that the existence of this maximum confirms the cluster structure of the alloy and is the result of the intercluster interference of X-rays [42].

The interatomic distance determined by the position of the maximum (R_1_ = 0.550 nm) can be considered as the average distance between the centers of clusters formed from rare earth elements (Y, Gd) and surrounded by Al atoms. The characteristic size of the clusters, estimated by the half-width of the maximum, reaches L ≈ 1.1 nm.

The main maximum is characterized by an asymmetrical shape, manifested in the existence of an influx from the side of larger scattering angles. To obtain quantitative structural information, the diffraction curve was approximated by superimposing individual maxima corresponding to scattering from structural units with different chemical compositions and/or types of short-range atomic ordering. The main maximum corresponds to scattering by clusters with an average interatomic distance R_2_ = 0.290 nm, which is close to the sum of the atomic radii of Al atoms (0.290 nm). At the same time, the appearance of a surge on the slope of the main maximum is caused by scattering from structural units with a significantly smaller interatomic distance R_3_ = 0.250 nm, which is less than the sum of the atomic radii of Al and Ni atoms (0.268 nm). Those clusters are formed with a predominant chemical interaction between the Al and Ni atoms. Estimated by the ratio of the integral intensity of the maxima, the volume fraction of chemically ordered regions reaches 30%.

**Figure 3:**
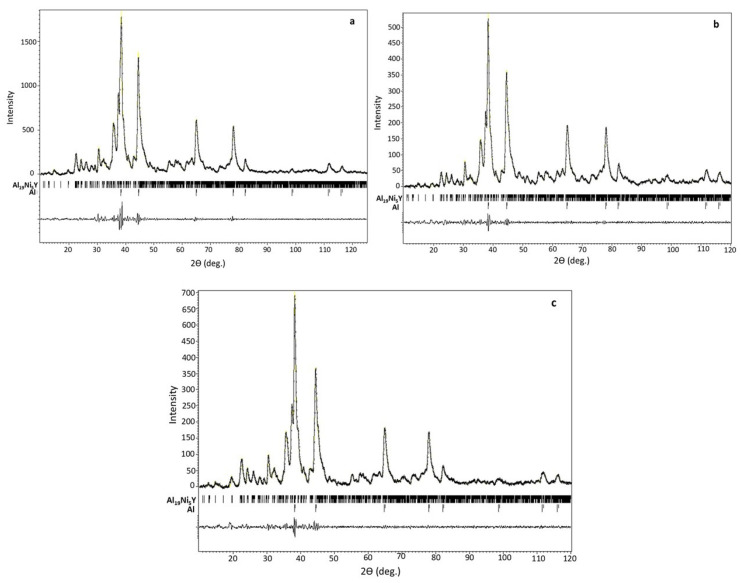
Diffractograms of annealed amorphous alloys Al_87_Y_4_Gd_1_Ni_8_ at (**a**) T_1_ = 624 ± 2 K, (**b**) T_2_ = 633 ± 2 K, (**c**) T_3_ = 643 ± 2 K for 2 min. The markers corresponding to both identified phases (Al_19_Ni_5_(Y,Gd)_3_ and Al) are generated in the form of a series visible in the drawing directly below the diffraction pattern along with the description.

During isothermal annealing of Al_87_Y_4_Gd_1_Ni_8_ alloys at temperatures (a) T_1_ = 624 ± 2 K, (b) T_2_ = 633 ± 2 K, and (c) T_3_ = 643 ± 2 K for 2 min, a crystalline structure is formed (Figure 3 and Table 3). In Figure 3, the alloy is characterized by a two-phase crystalline structure containing a solid solution based on Al (ICDD PDF 5+ 2026: 98-004-3423; cubic symmetry, Fm-3m space group) and the chemical compound Al_19_Ni_5_(Y,Gd)_3_ (ICDD PDF 5+ 2026: 98-016-0932; orthorhombic symmetry, Cmcm space group).

Table 3 shows the values of the parameters of the unit cell of the phase components and the average grain size calculated from the diffraction data. The lattice parameters of the Al (a ≈ 4.060 Å) are slightly larger than those of pure Al (a ≈ 4.050 Å), which indicates a slight solubility of the atoms of the doping elements. The grain sizes of the Al solid solution in samples annealed at T_1_ and T_2_ exceed 150–200 nm, and therefore their determination is beyond the scope of the X-ray scattering method.

However, there is a slight decrease in the size of Al grains in the sample annealed at T_3_ (L~120 nm), which can be explained by an increase in the grain nucleation rate with an increase in the annealing temperature. The sizes of the coherent scattering blocks of the Al_19_Ni_5_(Y,Gd)_3_ phase practically do not depend on the annealing temperature and vary in the range L~45–50 nm.

**Figure 4:**
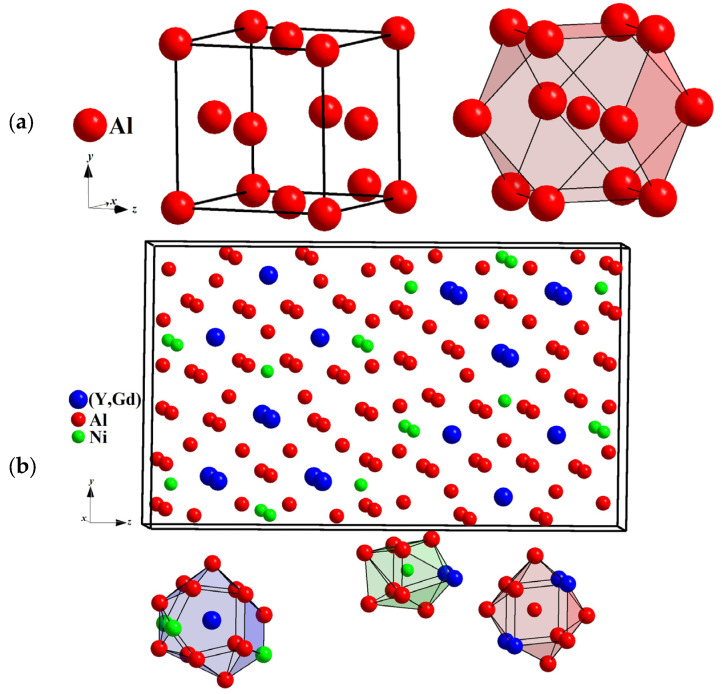
Unit cell and coordination polyhedrons for (**a**) Al atoms and (**b**) for Al, (Y,Gd), Ni atoms.

Figure 4 shows a visualized unit cell according to the data from Table 3. The coordination polyhedron for aluminum (Figure 4) atoms in the Cu-type structure (space group Fm-3m) is the cuboctahedron (coordination number CN = 12). The coordination polyhedron for biggest atoms (statistical mixture Y, Gd) in the Gd_3_Ni_5_Al_19_-type structure are 16-vertex Frank–Casper polyhedra with coordination number CN = 16, for the smallest atoms Ni—bicapped square prism CN = 10. For Al atoms the typical coordination number is 12, which represents a cuboctahedron or icosahedron.

### 4.2. TEM/HREM Analysis of Phases Transmission Within the Temperature Range (624–643) ± 1 K

**Figure 5:**
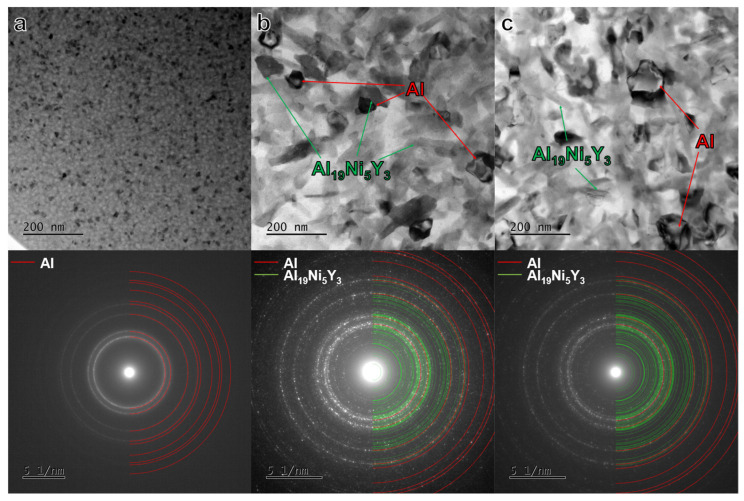
Bright-field TEM images of specimens annealed at (**a**) T_1_ = 624 ± 1 K, (**b**) T_2_ = 633 ± 1 K, and (**c**) T_3_ = 643 ± 1 K, together with their corresponding diffraction patterns.

**Figure 6:**
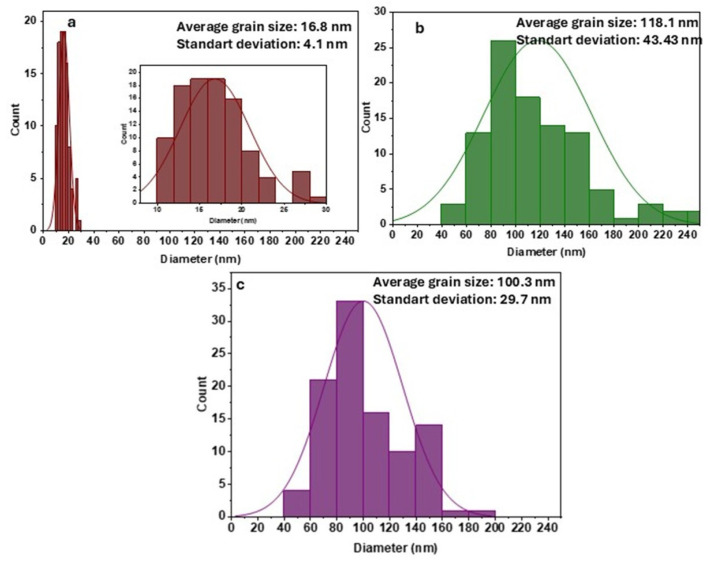
ED of annealed AMA Al_87_Y_4_Gd_1_Ni_8_ at (**a**) T_1_ = 624 ± 1 K, (**b**) T_2_ = 633 ± 1 K, (**c**) T_3_ = 643 ± 1 K for 2 min.

**Figure 7:**
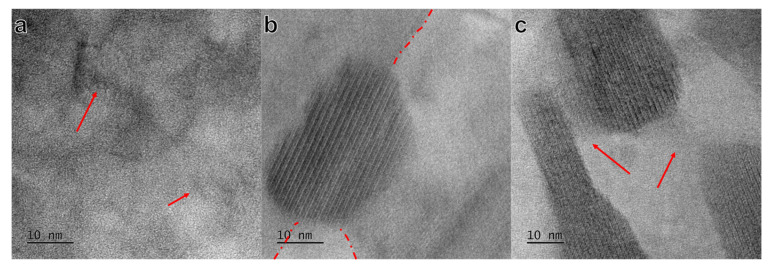
HRTEM images of specimens annealed at: (**a**) T_1_ = 624 ± 1 K, (**b**) T_2_ = 633 ± 1 K, and (**c**) T_3_ = 643 ± 1 K.

Samples annealed at temperatures T_1_ (624 ± 1 K), T_2_ (633 ± 1 K) and T_3_ (643 ± 1 K) were subjected to TEM observations. The observations showed that the T_1_ sample was fully crystalline, and no amorphous rings were observed in the diffraction pattern. Its structure consisted of the equiaxed grains of an Al solid solution (SS) with an average grain size of 16 ± 4 nm (Figure 5a and Figure 6a). Darker contrast can be observed at the grain boundaries (GBs) in the HREM images (Figure 7a). This may be attributed to GB segregation of Ni and RE elements due to their rejection during the growth of Al SS grains. This phenomenon was previously reported in the literature for Al–RE AMAs [43,44,45,46].

**Figure 8:**
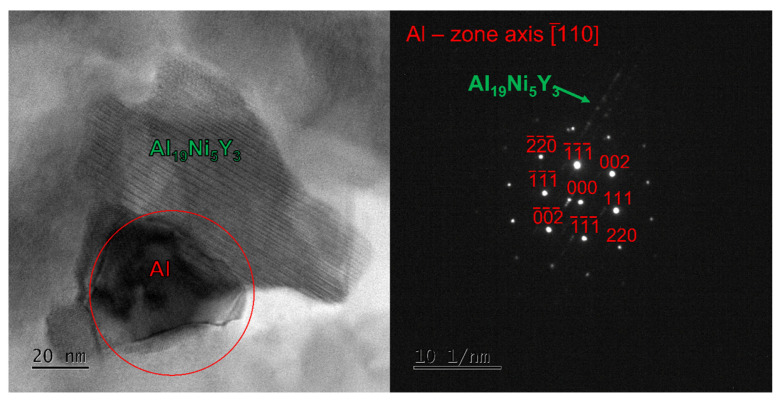
Bright-field TEM image of an Al SS grain in the T_2_ = 633 ± 1 K specimen showing two intermetallic grains formed along its grain boundaries.

The structure of the specimens annealed at T_2_ indicated that the Al SS grains grew substantially in an equiaxed manner (Figure 5b). However, a second phase, the Al_19_Ni_5_Y_3_ intermetallic, had formed. Smaller grains of this phase appeared to be equiaxed, whereas the larger grains were clearly more needle-like and appeared to be elongated predominantly along one direction. This phase appears to form and grow along the Al SS GBs (Figure 8). It was observed that equiaxed grains of Al_19_Ni_5_Y_3_ smaller than 60 nm were often located near GB triple-junction points (Figure 7b). This is in good agreement with the observed darker contrast at that area and the assumed RE enrichment in this region (red arrows).

The specimen annealed at T_3_ showed further growth of the Al SS grains, some of which exceeded 150 nm (Figure 6c). However, the apparent amount of this phase in the microstructure appeared to decrease. In contrast, the intermetallic phase became significantly coarser and emerged as the dominant constituent of the microstructure. All of its grains exhibited a needle-like morphology. Moreover, some of these grains appeared to coalesce at the expense of the Al SS phase (Figure 5c).

### 4.3. Changes in the Mechanical Properties of the AMA Al_87_Y_4_Gd_1_Ni_8_ Due to Phase Transition in the Temperature Range (624–643) ± 1 K

In our previus works [7,36] we present a studies of changes in microhardness due to annealing for the AMA Al_87_Y_4_Gd_1_Ni_8_ at different temperatures: 2.43 (510 K), and 2.05 (609 K). The microhardness values increase five times as a result of annealing at constant temperature T_3_ = 647 ± 1 K and 645 ± 1 K during 5 min. of annealing for the AMAs Al_87_Y_4_Gd_1_Ni_4_Fe_4_ and Al_87_Gd_5_Ni_4_Fe_4_, respectively, equalling 2 GPa.

**Figure 9:**
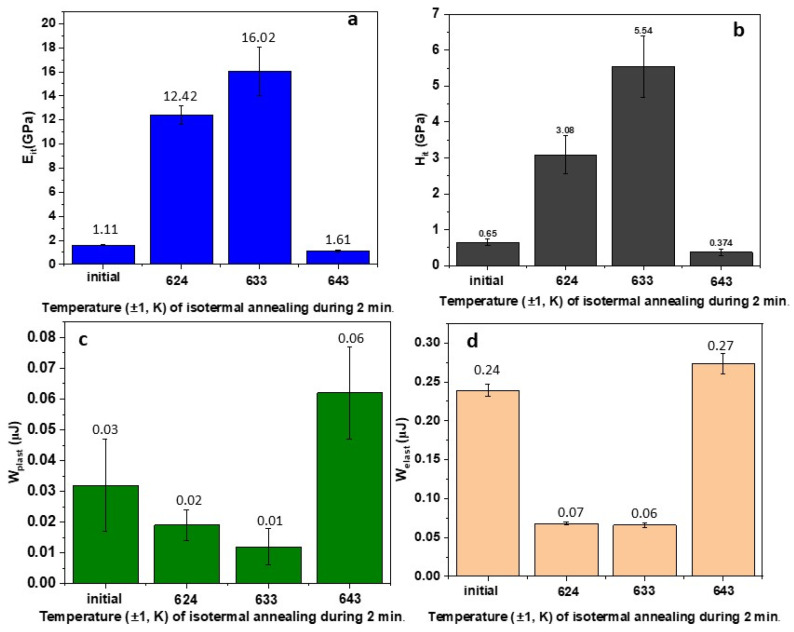
The dependences of the change in (**a**) indentation modulus (Oliver–Pharr method), (**b**) microhardness (Oliver–Pharr method), (**c**) plastic parts of the indentation work, (**d**) elastic parts of the indentation work on the annealing temperature of the AMA Al_87_Y_4_Gd_1_Ni_8_.

For the initial AMA Al_87_Y_4_Gd_1_Ni_8_, the microhardness is 0.6 ± 0.1 GPa (Figure 9b). Due to thermal annealing for 2 min. at T_1_ = 624 ± 1 K and T_2_ = 633 ± 1 K microhardness increases by five and eight times compared to the initial sample and equals 3.08 ± 0.53 GPa and 5.54 ± 0.85 Gpa, but the material is less elastic (Figure 9b–d).

The microhardness of a material characterizes the strength of the bonds between atoms in its structure; therefore, changes in microhardness indicate changes taking place in the structure of the amorphous phase. The increase in microhardness during annealing is associated both with processes involving a reduction in free volume and with processes of ordering. The optimal ratio of crystalline and amorphous phases provides maximum hardness. The amorphous phase acts as a “buffer” that prevents crack propagation, and nano-sized crystals block plastic deformation. According to the Hall–Petch effect [47]: In the range of grain sizes from tens to hundreds of nanometers, grain boundaries serve as obstacles to the movement of dislocations. Microhardness increases, but with decreasing grain size, microhardness decreases.

For this alloy, the optimal annealing at a temperature of 633 ± 1 K is to improve mechanical properties.

Table 4 shows the parameters of the maximum indentation depth. The error of this measurement is not significant and ranges from 0.07 to 0.1 μm for annealed AMA, which indicates uniformity in the crystallization process in amorphous matrix.

**Figure 10:**
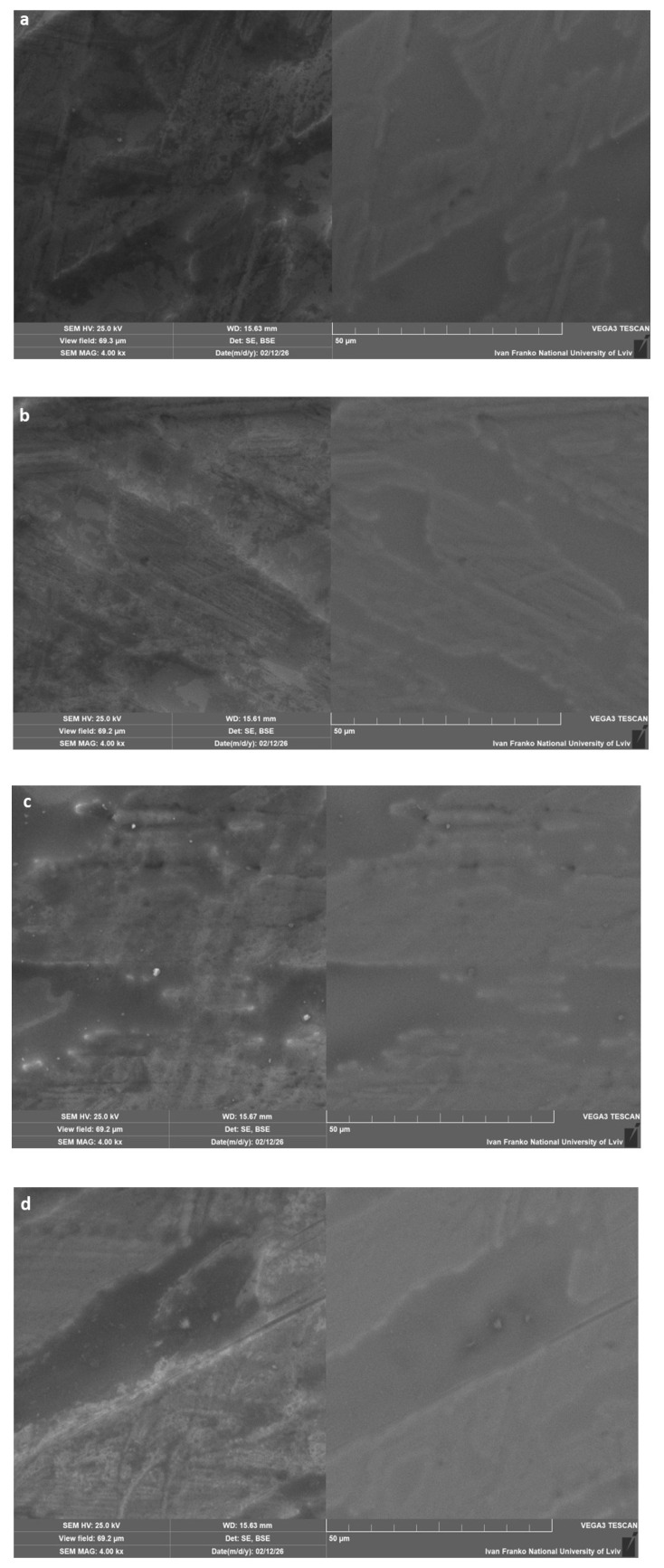
SEM images (×4000) of (**a**) initial AMA Al_87_Y_4_Gd_1_Ni_8_ and annealed at (**b**) T_1_ = 624 ± 1 K, (**c**) T_2_ = 633 ± 1 K, (**d**) T_3_ = 643 ± 1 K for 2 min.

**Figure 11:**
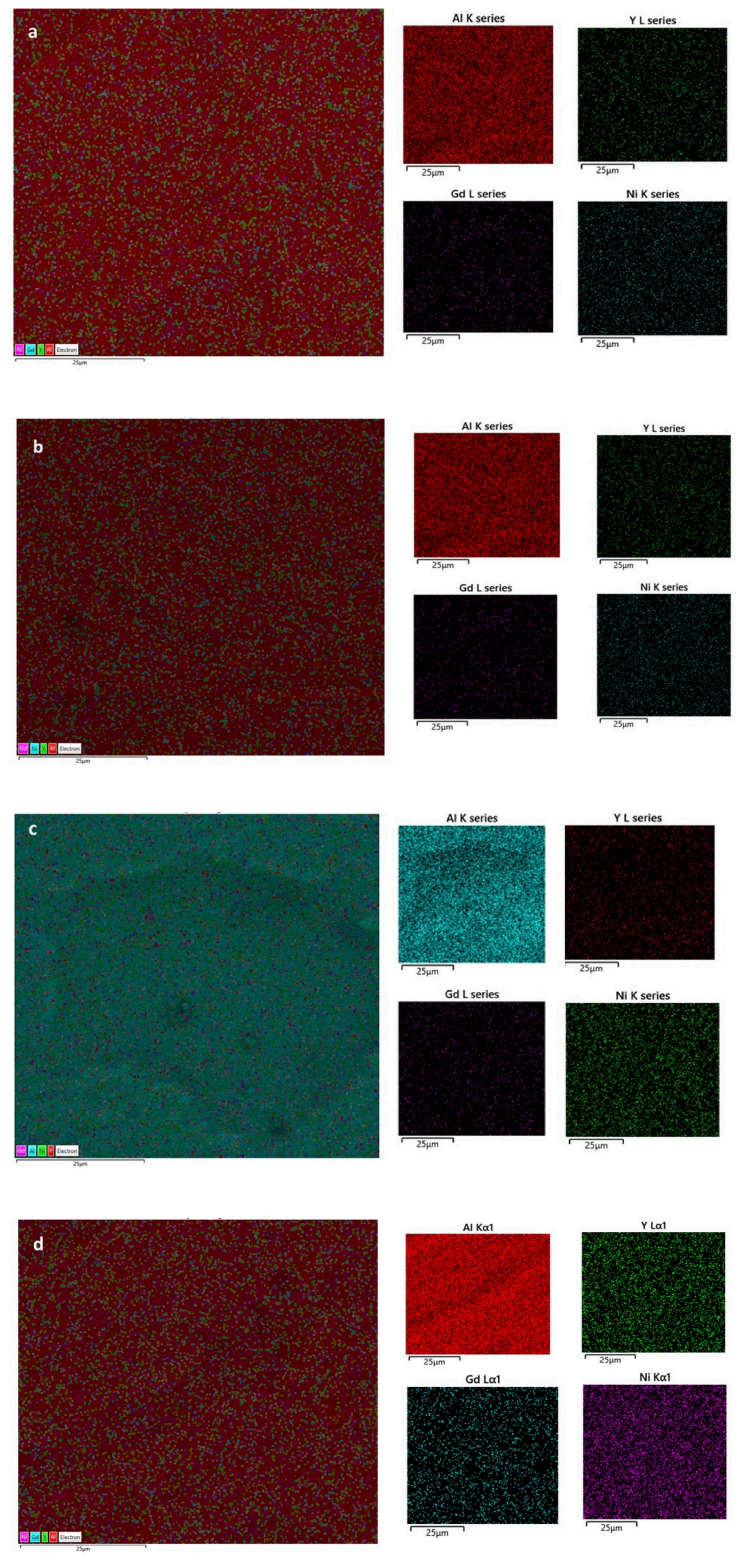
EDS images of (**a**) initial AMA Al_87_Y_4_Gd_1_Ni_8_ and annealed at (**b**) T_1_ = 624 ± 1 K, (**c**) T_2_ = 633 ± 1 K, (**d**) T_3_ = 643 ± 1 K for 2 min.

**Figure 12:**
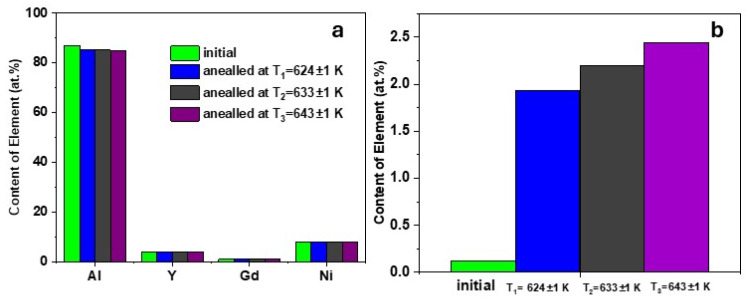
(**a**) The change in the content of elements on the AMA surface as a result of thermal modification at T_1_ = 624 ± 1 K, T_2_ = 633 ± 1 K, T_3_ = 643 ± 1 K for 2 min.; (**b**) Oxygen content on the AMAs surface as a result of thermal modification at T_1_ = 624 ± 1 K, T_2_ = 633 ± 1 K, T_3_ = 643 ± 1 K for 2 min.

Figure 10, Figure 11 and Figure 12 shows the results of SEM and EDS analyses of the surface of the initial and annealed AMA Al_87_Y_4_Gd_1_Ni_8_. The EDS method established that due to the increase in the annealing temperature, an increase in the oxygen content on the surface of AMA is observed, and a decrease in aluminum by ±2 at% is observed. Thus, numerous crystallites do not form on the surface, which indicates the formation of a natural but not uniform amorphous–crystalline oxide film [48]. The comparison between SEI and BSE images in Figure 10 is excellent, revealing better the microstructural aspects of the alloy surface.

Aluminum-based amorphous alloys exhibit exceptional ultimate tensile strengths typically ranging from 800 MPa to 1300 MPa [49,50]. The tensile strength for aluminum alloys in the amorphous state is approximately 800 MPa, and in the partially crystallized state after isothermal heat treatment −1500 MPa [51,52].

**Figure 13:**
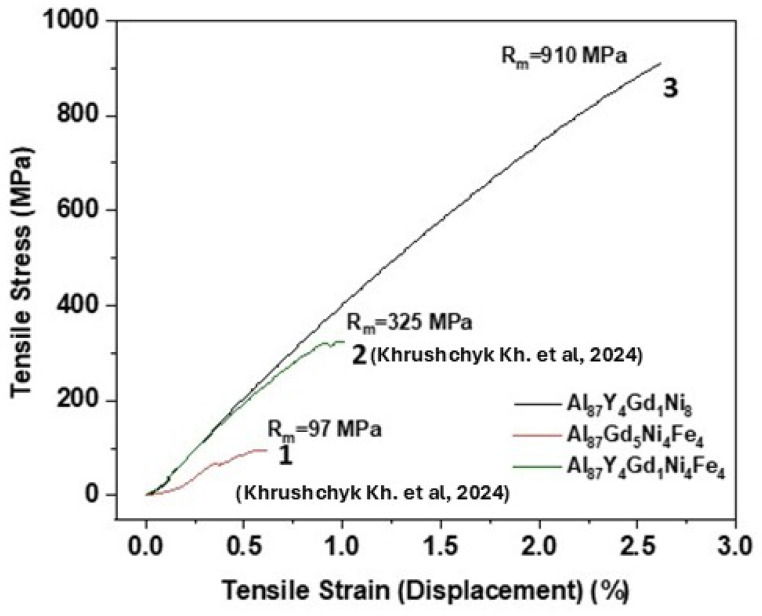
The graphs of the tensile test for initial AMAs: 1—Al_87_Gd_5_Ni_4_Fe_4_ [7]; 2—Al_87_Y_4_Gd_1_Ni_4_Fe_4_ [7]; 3—Al_87_Y_4_Gd_1_Ni_8_.

In our previous investigations [7] it was found that the tensile strength is 3.4 times higher for AMA Al_87_Y_4_Gd_1_Ni_4_Fe_4_ compared to Al_87_Gd_5_Ni_4_Fe_4_. Iron-containing AMAs based on aluminum of the composition Al–(Y,Gd)–(Ni,Fe) have tensile strength parameters (R_m_) of 325 MPa and 97 MPa for AMAs Al_87_Y_4_Gd_1_Ni_4_Fe_4_ and Al_87_Gd_5_Ni_4_Fe_4_, respectively (Figure 13). For the AMA Al_87_Y_4_Gd_1_Ni_8_ the parameter of tensile strength is 910 MPa, which is three times higher than for the AMA Al_87_Y_4_Gd_1_Ni_4_Fe_4_.

## 5. Conclusions

1. The activation energy of the nucleation, growth and stable crystallization processes and the frequency factor (k_0_) were calculated for the AMA Al_87_Y_4_Gd_1_Ni_8_. The activation energy of the nucleation (T_1_ = 624 ± 1 K), growth (T_2_ = 633 ± 1 K) and stable crystallization (T_3_ = 643 ± 1 K) processes equals to 193 ± 12, 199 ± 30, and 188 ± 18 kJ/mol and the frequency factor equals to 2.0 × 10^12^, 3.53 × 10^13^, and 4.41 × 10^11^ s^−1^, respectively.
2. It was established that during isothermal annealing in the temperature range of 624–643 K, a thermally stable compound Al_19_Ni_5_(Y,Gd)_3_ was formed.
3. Using the energy-dispersive spectroscopy method it was determined that as the annealing temperature increases, the oxygen content on the surface of the AMA rises while the aluminum content decreases by ±2 at.%, which may indicate the formation of a heterogeneous amorphous–crystalline oxide film.
4. Mechanical properties are improved by five and nine times after annealing at the temperatures of nucleation (T_1_ = 624 ± 1 K) and crystal growth (T_2_ = 633 ± 1 K) compared to the initial state of the amorphous metal sample and equal 3.08 ± 0.53 GPa and 5.54 ± 0.85 GPa. Annealing at the temperatures of stable crystallization (T_3_ = 643 ± 1 K) sharply worsens this property of the material.
5. For the AMA Al_87_Y_4_Gd_1_Ni_8_ the parameter of tensile strength is 910 MPa, which is three times higher than for the AMA Al_87_Y_4_Gd_1_Ni_4_Fe_4_.

## Figures and Tables

**Table 1:** A comparison of the influence of iron atoms on phase transition temperatures, activation energies, and identifiable phases.

System of AMA	T_a_, K	Number of Phase Transition Stages	Activation Energy, kJ/mol	Identified Phases
Al–Y(Gd)–Ni	615–630	1–3	~160–200	Al, Al_3_Y, Al_3_Gd, Al_3_Ni, Al_23_Ni_6_Y_4_, Al_19_Ni_5_Y_3_
Al–Y(Gd)–Ni–Fe	600–670	3–5	~150–250	Al, AlFe, Al_3_Ni, Al_13_Fe_4_, Al_23_Ni_6_Y_4_, Al_10_Fe_2_Y, Al_19_Ni_5_Y_3_ Al_15_Fe_9_Y_2_

**Table 2:** Structure parameters of amorphous metal alloy Al_87_Y_4_Gd_1_Ni_8_.

Amorphous Phase	2θ, °	S, nm^−1^	I/I_0_	FWHM	R, nm	L, nm
Ph1	19.5	14.0	7.7	5.87	0.550	1.1
Ph2	38.1	27.0	100	3.96	0.290	1.6
Ph3	43.8	31.0	33.7	5.53	0.250	1.1

2θ—the angular position of the maxima; S—the modulus of the wave vector corresponding to the maximum scattering intensity; I/I_0_—the relative scattering intensity; FWHM—the angular half-width of the maximum; L—the size of the coherent scattering regions; R—the average interatomic distance.

**Table 3:** Structure parameters of AMA Al_87_Y_4_Gd_1_Ni_8_ after isothermal annealing during 2 min at T_1_ = 624 ± 1 K, T_2_ = 633 ± 1 K, T_3_ = 643 ± 1 K.

Temperatures of Annealing ± 1, K	Phase Composition	Space Group	Parameters of the Crystalline Cell, Å	L, nm
624	Al	Fm-3m	4.0604 ± 0.0005	>150–200
	Al_19_Ni_5_(Y,Gd)_3_	Cmcm	4.0713 ± 0.0007	~45–50
			16.007 ± 0.0031	
			26.932 ± 0.0058	
633	Al	Fm-3m	4.0622 ± 0.0004	>150–200
	Al_19_Ni_5_(Y,Gd)_3_	Cmcm	4.0806 ± 0.0004	~45–50
			16.0040 ± 0.0018	
			27.0060 ± 0.0035	
643	Al	Fm-3m	4.0597 ± 0.0005	~120
	Al_19_Ni_5_(Y,Gd)_3_	Cmcm	4.0787 ± 0.0006	~45–50
			15.9826 ± 0.0023	
			26.9840 ± 0.0048	

L—the size of the coherent scattering regions.

**Table 4:** The values of maximum indention depth (µm) parameters for the initial and annealed AMA Al_87_Y_4_Gd_1_Ni at T_1_ = 624 ± 1 K, T_2_ = 633 ± 1 K, T_3_ = 643 ± 1 K for 2 min.

Temperature of Annealed ± 1, K	h_max_ (µm)
initial	5.324 ± 0.084
624	2.075 ± 0.085
633	1.765 ± 0.100
643	6.511 ± 0.072

## Data Availability

The original contributions presented in this study are included in the article. Further inquiries can be directed to the corresponding author.

## Abbreviations

The following abbreviations are used in this manuscript:

AMA	Amorphous metal alloys
DSC	Differential Scanning Calorimetry
XRD	X-ray diffraction
TEM	Transmission Electron Microscopy
HREM	High-Resolution Electron Microscopy analysis
REE	Rare-earth element
GFA	Glass-forming ability
TM	Transition Metal
